# Phytochemical Traits and Biological Activity of *Satureja hortensis* and *Satureja macrantha* as Culinary Spices Using GC–MS/MS and LC–MS/MS Techniques

**DOI:** 10.1002/fsn3.70733

**Published:** 2025-08-13

**Authors:** Pardis Elmdoustazar, Bilge Aydın, Mehmet Önal, Hafize Yuca, Mehmet Karadayı, Yusuf Gülşahin, Betül Demirci, Songül Karakaya, Zühal Güvenalp

**Affiliations:** ^1^ Faculty of Pharmacy, Department of Pharmacognosy Atatürk University Erzurum Türkiye; ^2^ Faculty of Pharmacy, Department of Pharmacognosy Erzincan Binali Yıldırım University Erzincan Türkiye; ^3^ Eastern Anatolia Forestry Research Institute Erzurum Türkiye; ^4^ Faculty of Science, Department of Biology Atatürk University Erzurum Türkiye; ^5^ Faculty of Pharmacy, Department of Pharmacognosy Anadolu University Eskişehir Türkiye; ^6^ Faculty of Pharmacy, Department of Pharmaceutical Botany Atatürk University Erzurum Türkiye

**Keywords:** anticholinesterase, antidiabetic, antimicrobial, antioxidant, genotoxicity, *Satureja*

## Abstract

This study evaluates the antidiabetic, anti‐Alzheimer, antimicrobial, and antioxidant activities of water and methanol extracts, as well as essential oils, from *Satureja macrantha* and *Satureja hortensis* . Chemical characterization was conducted using LC–MS/MS for phenolic profiles and GC–MS for essential oil composition. Biological activities were assessed via DPPH˙ and ABTS˙^+^ assays for antioxidant capacity, α‐glucosidase and α‐amylase inhibition for antidiabetic potential, cholinesterase inhibition for anti‐Alzheimer activity, and the microdilution method for antimicrobial effects. Genotoxicity and antigenotoxicity were evaluated using Ames and *Allium cepa* assays. Rosmarinic acid was most abundant in *S. macrantha* water extract (50.687.02 ng/mL), while thymol (45.1%) and p‐cymene (45.8%) were identified as major oil components. *S. hortensis* essential oil showed the strongest α‐glucosidase (41.33%) and butyrylcholinesterase inhibition (35.68%), whereas *S. macrantha* oil exhibited higher acetylcholinesterase inhibition (16.05%). Antioxidant activity was most pronounced in *S. macrantha* water extract (ABTS^˙+^: 52.11%, DPPH˙: 28.76%). *S. hortensis* essential oil demonstrated the most effective antimicrobial properties. These findings suggest that both *S. macrantha* and *S. hortensis* could be recommended as safe and effective candidates for use in the development of natural therapeutic agents.

## Graphical abstract

Antidiabetic, anti‐Alzheimer, antimicrobial, and antioxidant activities of *Satureja macrantha* and *Satureja hortensis* extracts and oils. p‐Cymene and thymol were the major components found in *S. macrantha* and *S. hortensis*, respectively. *S. hortensis* essential oil showed notable α‐glucosidase inhibition (41.33%) and anticholinesterase activity. *S. macrantha* water extract had the highest antioxidant activity, with strong ABTS˙^+^ and DPPH˙ scavenging effects. Both species demonstrated promising biological activities with no genotoxic effects, supporting their safe use.

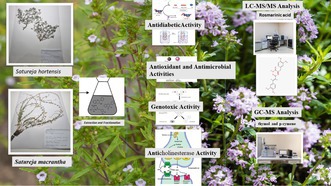

## 1 Introduction

Diabetes mellitus (DM) is a chronic metabolic disorder marked by sustained hyperglycemia resulting from insufficient insulin secretion or impaired insulin utilization. Affecting individuals across all demographics and regions, it is a leading global cause of morbidity and mortality. Type 2 diabetes accounts for over 90% of cases and arises from a combination of genetic predisposition and environmental influences. The two primary forms of DM—type 1 and type 2—have well‐established diagnostic criteria (Hossain et al. 2024).

Alzheimer's disease (AD) is a neurodegenerative disorder that causes memory loss and brain shrinkage, leading to dementia. Diagnosis often takes 2.8–4.4 years after symptoms begin. Early detection is critical, especially in early‐onset cases. In 2015, 46.8 million people had dementia globally—a number projected to reach 131.5 million by 2050. Early AD involves amyloid β accumulation and tau pathology, with genetics accounting for 60%–80% of risk—particularly APOE alleles and over 40 related genes. Emerging biomarkers like PET imaging and plasma tests show promise for early detection. While lifestyle changes may support cognition, they do not alter disease progression. Current pharmacological trials targeting amyloid β, tau, and inflammation offer hope for future therapies (Scheltens et al. 2021; Hafeez et al. 2025).

Recent findings reveal that obesity and its comorbidities, such as type 2 diabetes mellitus (T2DM), share common molecular mechanisms with AD, including insulin resistance and impaired insulin signaling. These shared pathways—along with oxidative stress, neuroinflammation, and disrupted energy balance—contribute to neurodegeneration. Neuropeptide metabolism, crucial for appetite and energy regulation, is also altered in both metabolic and neurodegenerative disorders. As a result, treatments developed for obesity or T2DM may offer therapeutic potential for AD, particularly those targeting insulin and leptin pathways (Mengr et al. 2025).

Reactive oxygen species (ROS) formed during normal metabolism can lead to oxidative stress when not balanced by antioxidants. This imbalance is associated with chronic diseases, making plant‐based antioxidants a promising approach for protection and redox balance (Belew and Gebre 2025). Oxidative stress (OS), often called the “chemical silent killer,” progresses without symptoms and lacks a direct diagnostic test. It plays a central role in the onset and progression of many diseases. Antioxidants—produced by the body or obtained from diet—help counteract OS by protecting biomolecules from oxidative damage (Arınmış et al. 2024).

Plants synthesize a diverse array of natural compounds known as secondary metabolites, which are produced via pathways distinct from those of primary metabolism. These bioactive compounds hold significant value in various sectors, including pharmaceuticals, cosmetics, and food industries (Elbouzidi et al. 2024). For centuries, medicinal plants have been widely utilized not only in traditional medicine but also in areas such as food preservation. Today, their relevance continues to grow in both the pharmaceutical and food industries, especially due to the demand for safe, natural, and eco‐friendly solutions. Notably, aromatic plants have also attracted attention in the field of animal nutrition, where they are expected to contribute significantly as functional feed additives (Giannenas et al. 2020).

*Satureja hortensis* L., commonly known as summer savory, is an annual, herbaceous plant belonging to the Lamiaceae (Labiatae) family. It emits a thyme‐like aroma and typically grows to a height of 10–30 cm, with numerous branched stems. The leaves are narrow, pointed, sessile, and covered with fine hairs, measuring approximately 1–3 cm in length and up to 0.5 cm in width. The plant bears small white or pink flowers with a bilabiate corolla. The calyx is composed of five sharp teeth, nearly equal in length to the tubular portion. The fruits are small, capsule‐like, and brown to black in color. Its seeds are very tiny, with a thousand‐seed weight of approximately 0.5–0.6 g (Çeri 2022). Aromatic perennial herb, 20–50 cm tall. Stems are erect or spreading, branched at the base, with short retrorse hairs. Leaves are sessile, linear to oblanceolate, 10–18 mm long and 2–5 mm wide, often folded, with sparse rough hairs and sessile glands. Flowers are arranged in verticillasters with 1–6 flowers, mostly sessile. Calyx is tubular, green or rarely purple, 4–6 mm long, with slightly recurved teeth and scattered hairs and glands. Corolla is violet with a white tube, 12–15 mm long. Stamens slightly protrude; the style is similar in length to posterior stamens. Nutlets are elliptic, 1.2–1.5 mm long, brown to dark brown (Bordbar et al. 2025). *S. macrantha* is used in Iran primarily as a food sweetener and for treating urinary tract diseases (Aghbash et al. 2020). *S. hortensis* (summer savory) is a widely used herb, especially in meat‐based dishes such as poultry, stews, and sausages. It is a key ingredient in traditional spice blends and recipes across Eastern Europe and Canada. Often available in dried form, it adds a rich aroma to various meals. Besides its culinary use, it has also been traditionally used for digestive ailments and is known for its antibacterial and antioxidant properties (Ejaz et al. 2023). Summer savory has long been used in traditional medicine to relieve cramps, muscle pain, nausea, digestive issues, diarrhea, and infections. Its essential oil exhibits notable antioxidant, antibacterial, and antifungal properties. The plant is also widely utilized in the food, beverage, and fragrance industries worldwide (Katar 2022).

Summer savory ( *S. hortensis* ), native to the Mediterranean and southern Europe, is a culinary and medicinal herb traditionally used for its aromatic leaves. Rich in volatile oils, phenolic acids, flavonoids, and tannins, it has shown potential benefits in managing diabetes, cardiovascular diseases, cancer, and Alzheimer's, alongside antioxidant, antimicrobial, and anti‐inflammatory properties. Historical records trace its use back to 183 bc in Roman northern Italy. Widely used in European cuisine—especially in meat dishes, stews, and herbal blends like Herbes de Provence—it is valued for aiding digestion and enhancing flavor. With a long history of safe use, *S. hortensis* is regarded as safe for regular consumption (Fierascu et al. 2018). *S. macrantha* is a small, branched shrub with hairy stems and narrow leaves, native to western and northwestern Iran. Traditionally used in Iranian folk medicine, it has been applied to treat diarrhea, wounds, gastroenteritis, and infections of the respiratory and urinary tracts. Its essential oil is rich in bioactive compounds such as p‐cymene, thymol, carvacrol, limonene, γ‐terpinene, and spathulenol (Nezhadasad Aghbash et al. 2021). Essential oils from medicinal plants such as *Satureja* species show promise as natural food preservatives. *S. macrantha* , native to western and northwestern Iran, is traditionally used both as a culinary herb and for its medicinal benefits, especially in managing urinary disorders (Aghbash et al. 2020).

This study investigates and compares the essential oil compositions and phenolic profiles of *S. hortensis* and *S. macrantha* , two medicinal plants traditionally used in Türkiye and Iran. The research further evaluates their antimicrobial, anticholinesterase, antioxidant, and antigenotoxic activities, as well as their inhibitory effects on α‐glucosidase and α‐amylase enzymes. Methanol and water extracts, along with essential oils obtained from the flowering aerial parts of both species, were analyzed for their phenolic constituents using LC–MS/MS, while essential oil profiles were determined by GC–MS.

## 2 Material and Methods

### 2.1 Plant Material

The aerial parts of *Satureja hortensis* were collected in the morning hours of mid‐June 2021 from Sülünkaya Village, Oltu District, Erzurum Province, Türkiye (41°44′44.42″ N, 41°57′03.80″ E) by Prof. Dr. Zühal Güvenalp and Forest Engineer Dr. Mehmet Önal. The plant was taxonomically identified and confirmed by Dr. Mehmet Önal from the Erzurum Regional Directorate of Forestry. Immediately after collection, plant materials were carefully cleaned to remove dust, soil, and other contaminants. The samples were then air‐dried in a shaded, well‐ventilated area at room temperature for 7–10 days to prevent degradation of active compounds. After drying, the plant materials were ground to a coarse powder using a laboratory mill and stored in airtight containers at room temperature in the dark until further analysis.

Similarly, *Satureja macrantha* was collected during the morning in the last week of July 2023 from the Darreh‐ye Qasemlu region, Urmia, Iran (37.3462° N, 45.1504° E) by Pardis Elmdoustazar. The species was identified and authenticated by Dr. Mojgan Larti, Shahram Bahadori, and Dr. Farhad Frozin. Post‐harvest procedures, including cleaning, shade drying, grinding, and storage, were performed in the same manner as described for *S. hortensis* .

Voucher specimens of both plant species were deposited at the Atatürk University Biodiversity Science Museum Herbarium with accession numbers ATA 415 ( *S. hortensis* ) and ATA 418 ( *S. macrantha* ), respectively.

### 2.2 Extraction

For the extraction process, 30 g of dried and powdered aerial parts of *S. hortensis* and 50 g of dried and powdered aerial parts of *S. macrantha* were used. The plant materials were collected during the flowering stage, which represents the optimal phenological period for secondary metabolite accumulation. Each plant material was separately subjected to maceration using methanol as the extraction solvent. The maceration was performed at room temperature (approximately 22°C–25°C) for three consecutive days, with the plant material immersed in fresh methanol (1:10 w/v) for 8 h per day under occasional stirring. At the end of each daily extraction cycle, the mixtures were filtered through Whatman No. 1 filter paper to separate the solid residues. The filtrates obtained from each day were combined and subjected to solvent removal under reduced pressure using a rotary evaporator (Büchi Rotavapor) at a temperature not exceeding 40°C, in order to preserve bioactive compounds. The resulting concentrated crude extracts were weighed, transferred into airtight, amber‐colored containers, and stored at +4°C in the dark until further phytochemical and bioactivity analyses.

In addition to methanol extraction, 15 g of dried and powdered aerial parts of each plant species ( *S. hortensis* and *S. macrantha* ) were used to prepare water extracts via decoction. The plant material was added to distilled water (1:20 w/v) and boiled gently for 30 min under continuous stirring to ensure effective extraction of water‐soluble constituents. Following decoction, the mixtures were allowed to cool to room temperature and then filtered separately using Whatman No. 1 filter paper to remove plant residues. The clear aqueous extracts were then frozen at −80°C for 24 h and subsequently lyophilized using a laboratory freeze‐dryer (lyophilizer) to obtain dry powder extracts. The final yields were accurately weighed and stored in airtight containers at +4°C until further use in analytical and bioactivity studies.

Methanol and water were selected as extraction solvents due to their wide polarity range and their proven efficacy in extracting a broad spectrum of bioactive compounds, particularly phenolics and flavonoids. Methanol is commonly used in phytochemical studies for its efficiency in extracting both moderately polar and polar compounds, while water, as a universal solvent, is especially important for simulating traditional preparation methods such as herbal infusions and decoctions. Moreover, the use of these two solvents allows for a comparative evaluation of biologically relevant compounds under both laboratory‐based and traditional conditions.

### 2.3 Essential Oil Extraction

Essential oils were extracted by hydrodistillation using a Clevenger‐type apparatus for 4 h. A total of 206.5 g of dried flowering aerial parts of *S. macrantha* and 250 g of *S. hortensis* were used. Distilled water was added in sufficient volume to fully immerse the plant materials, ensuring optimal recovery of volatile constituents.

### 2.4 GC‐FID Analysis

The GC analysis was carried out using an Agilent 7890B GC System. FID detector temperature was 250°C. Relative percentage amounts of the separated compounds were calculated from FID chromatograms.

### 2.5 GC–MS Analysis

The GC–MS analysis was carried out with an Agilent 7890B GC 5977B Mass Selective Detector System. Innowax FSC column (60 m × 0.25 mm, 0.25 μm film thickness) was used with helium as carrier gas (0.7 mL/min). GC oven temperature was kept at 60°C for 10 min and programmed to 220°C at a rate of 4°C/min, kept constant at 220°C for 10 min, and then programmed to 240°C at a rate of 1°C/min, for a total of 80 min. Split ratio was adjusted at 40:1. The injector temperature and ion source temperature were set at 250°C and 230°C, respectively. Mass spectra were recorded at 70 eV. Mass range was from *m/z* 35 to 450.

### 2.6 Identification of Components

Essential oil components were identified by comparing their relative retention times and retention indices (RRI) with those of authentic standards and a series of n‐alkanes. Identification was further supported by mass spectral matching using commercial libraries (Wiley 9 and NIST 11) and an in‐house database, the “Başer Library of Essential Oil Constituents,” which includes spectra of genuine compounds and well‐characterized essential oil components.

### 2.7 Qualitative and Quantitative Analysis of Secondary Metabolites

Qualitative and quantitative analysis of secondary metabolites in methanol and water extracts was conducted using an Agilent 6460 Triple Quadrupole LC–MS/MS system at Atatürk University's Eastern Anatolian High Technology Application and Research Center (DAYTAM). Chromatographic separation was achieved on an Agilent 1260 HPLC system equipped with a Poroshell 120 EC‐C18 column (4.6 × 100 mm, 3.5 μm) using electrospray ionization (ESI) in positive mode. Standard compounds were prepared in methanol (100 μg/mL) and serially diluted to construct calibration curves. The gradient elution program was as follows: 0–4 min, 5% B; 4–7 min, 20% B; 7–14 min, 90% B; 14–15 min, hold at 90% B; 15–15.1 min, decrease to 5% B; and 15.1–17 min, re‐equilibration at 5% B. The flow rate was 0.4 mL/min with a 5 μL injection volume, and total run time was 17 min. Mass spectrometric parameters were optimized as follows: nitrogen gas temperature at 350°C, flow rate 12 L/min; carrier gas temperature at 250°C, flow rate 5 L/min; nebulizer pressure at 55 psi. Data were acquired using Agilent MassHunter software, with a full scan range of m/z 50–1300.

### 2.8 Total Phenolic Content Quantification

The total phenolic content (TPC) of the water and methanol extracts of *Satureja hortensis* and *Satureja macrantha* was determined using the Folin–Ciocalteu method, as described by Singleton and co‐workers (Folin and Denis 1912; Slinkard and Singleton 1977). Results were expressed as gallic acid equivalents (GAE). A stock solution of gallic acid (1 mg/mL) was prepared and serially diluted to obtain standard concentrations ranging from 100 to 700 μg/mL. Folin–Ciocalteu reagent and sodium carbonate (Na₂CO₃) were added to each standard and sample solution, and absorbance was measured at 760 nm against a blank (distilled water). A calibration curve was generated using the equation: Absorbance = 0.0012 × GAE + 0.0304. The TPC of each extract (1 mg/mL) was calculated by substituting their absorbance values into the calibration equation. All measurements were conducted in triplicate to ensure reliability and reproducibility.

### 2.9 Total Flavonoid Content Quantification

The total flavonoid content in the water and methanol extracts of *S. hortensis* and *S. macrantha* was determined using a method based on Liu et al. (2007), with slight modifications. A flavonoid standard compound was dissolved in dimethyl sulfoxide (DMSO) to achieve a concentration of 1 mg/mL. From this stock solution, a series of working solutions with varying concentrations was prepared. Following the method of Nikolaeva et al. (2022), these solutions were treated with Folin–Ciocalteu reagent (FCR) and sodium carbonate (Na₂CO₃). Absorbance values were recorded at specific concentrations, and a standard calibration curve was constructed. The total flavonoid content of the extracts was calculated as a percentage based on the routine standard compound and expressed in μg rutin equivalent (RE) per mg of extract. All measurements were performed in triplicate to ensure the reliability and accuracy of the results.

### 2.10 Total Tannin Content Quantification

The total tannin content of the water and methanol extracts of *Satureja hortensis* and *Satureja macrantha* was determined using the Folin–Ciocalteu method, as modified by Makkar (2003). Tannic acid was used as the reference standard, with calibration solutions prepared in the range of 100–600 μg/mL. Extracts were prepared at a concentration of 1 mg/mL. Each standard and extract sample was mixed with 0.5 mL of Folin–Ciocalteu reagent and 1.5 mL of 2% sodium carbonate (Na₂CO₃) solution. After incubation, absorbance was measured at 725 nm against a distilled water blank. Tannin content was calculated from the standard curve and expressed as tannic acid equivalents (TAE, μg). All measurements were performed in triplicate to ensure accuracy and reproducibility.

### 2.11 ABTS Cation Radical Scavenging Capacity

The ABTS˙^+^ scavenging capacities of *S. hortensis* and *S. macrantha* extracts were evaluated using the method developed by Re et al. (1999). In this assay, α‐tocopherol and trolox were used as standards to test their antioxidant effects against a 2 mM ABTS˙^+^ solution. First, the percentage inhibition values of the standards against ABTS˙^+^ at concentrations ranging from 1 to 100 μg/mL were measured to determine the concentration range for testing the extracts. Serial dilutions of the extracts were prepared in the concentration range of 10–100 μg/mL, and their antioxidant activities were assessed by measuring the inhibition of ABTS˙^+^ at 734 nm, using phosphate buffer as the blank. The ABTS˙^+^ scavenging capacity of the extracts was calculated based on the following equation: % Inhibition = [(*A*_control_ − *A*_sample_)/*A*_control_] × 100.

### 2.12 DPPH Radical Scavenging Capacity

The DPPH˙ scavenging capacities of *S. hortensis* and *S. macrantha* extracts were assessed using the method described by Blois (1958). In this assay, α‐tocopherol and trolox were used as standards to evaluate their antioxidant effects against a 1 mM DPPH˙ solution. Initially, the percentage inhibition values of the standards were determined at concentrations ranging from 1 to 100 μg/mL, and based on these values, the concentration range for testing the extracts was decided. Serial dilutions of the extracts, ranging from 10 to 100 μg/mL, were prepared, and their antioxidant activities were measured by evaluating the inhibition of DPPH˙ at 517 nm, using 99% ethanol as the blank. The DPPH˙ scavenging capacity of the extracts was calculated using the following formula: % Inhibition = [(*A*_control_ − *A*_sample_)/*A*_control_] × 100.

### 2.13 α‐Glucosidase Inhibitory Activity Determination

The α‐glucosidase inhibition assay was carried out with modifications based on the method of Bachhawat et al. (2011). α‐Glucosidase (0.1 U/mL) was prepared in 0.1 M potassium phosphate buffer (pH 6.9). Extracts and sub‐extracts of *Satureja hortensis* and *Satureja macrantha* were dissolved in DMSO at concentrations ranging from 1 to 5000 μg/mL. The assay was performed in 96‐well microplates by adding 50 μL of buffer, 10 μL of enzyme solution, and 20 μL of extract. After incubation at 37°C for 5 min, 20 μL of 3 mM *p*‐nitrophenyl‐α‐D‐glucopyranoside was added as the substrate. The mixture was then incubated at 37°C for 30 min. The reaction was terminated by adding 50 μL of 0.1 M sodium carbonate, and the absorbance of the released *p*‐nitrophenol (pNP) was measured at 405 nm. Acarbose was used as a positive control. Results were expressed as percentage inhibition relative to the control, calculated using the formula: %Inhibition = [(*A*_control_ − *A*_sample_)/*A*_control_] × 100.

### 2.14 α‐Amylase Enzyme Inhibition Determination

The α‐amylase inhibitory activity was evaluated following the method of Nampoothiri et al. (2011) with minor modifications. Test samples were prepared at concentrations ranging from 1 to 5000 μg/mL. Each sample (100 μL) was mixed with 100 μL of 1% starch solution prepared in 20 mM sodium phosphate buffer (pH 6.9, containing 6 mM NaCl) and incubated at 25°C for 10 min. Subsequently, 100 μL of porcine pancreatic α‐amylase (0.5 mg/mL) was added, and the mixture was incubated for another 10 min at 25°C. The reaction was terminated by adding 200 μL of dinitrosalicylic acid (DNS) reagent, followed by heating at 100°C for 5 min. After cooling to room temperature, 50 μL of each reaction mixture was transferred to a new 96‐well plate and diluted with 200 μL of distilled water. Absorbance was measured at 540 nm using a microplate reader. Acarbose was used as a positive control. The percentage inhibition of α‐amylase activity was calculated using the formula: %Inhibition = [(*A*_control_ − *A*_sample_)/*A*_control_] × 100.

### 2.15 Acetylcholinesterase (AChE) and Butyrylcholinesterase (BChE) Inhibition Assays

The enzyme inhibitory activities of acetylcholinesterase (AChE) and butyrylcholinesterase (BChE) were evaluated using the method described by Ingkaninan et al. (2000), with slight modifications. In a 96‐well microplate, 125 μL of 5,5′‐dithiobis‐(2‐nitrobenzoic acid) (3 mM, DTNB, Ellman's Reagent), 25 μL of the respective substrate (15 mM acetylthiocholine iodide for AChE and 15 mM butyrylthiocholine iodide for BChE), 50 μL of Tris–HCl buffer (50 mM, pH 8), and 25 μL of the sample were added. Following this, 25 μL of the respective enzyme (AChE or BChE) was introduced to initiate the reaction. The mixtures were incubated for 10 min for the AChE inhibition assay and for 15 min for the BChE inhibition assay at 37°C. The reaction progress was measured spectrophotometrically at 405 nm, and donepezil was used as a positive control. The inhibition percentage for both enzymes was calculated using the formula below: %Inhibition = [(*A*_control_ − *A*_sample_)/*A*_control_] × 100.

### 2.16 Antimicrobial Activity

Antimicrobial activity was evaluated using the Minimum Inhibitory Concentration (MIC) method. The antimicrobial effect was tested against a variety of bacterial strains, including *Methicillin‐resistant Staphylococcus aureus* (ATCC 67106), *Acinetobacter baumannii* (ATCC BA1609), *Enterococcus faecium* (ATCC 700211), *Escherichia coli* (ATCC BAA‐2523), *Streptococcus salivarius* (ATCC 13419), *Pseudomonas aeruginosa* (ATCC 9070), *Streptococcus mutans* (ATCC 35668), *Enterococcus faecalis* (ATCC 49452), and *Bacillus cereus* (ATCC 14579).

### 2.17 Disk Diffusion Test

The disk diffusion assay was performed to evaluate the antimicrobial activity of the test substances. Stock solutions (up to 30 mg/mL) were prepared in appropriate solvents and sterilized using a 0.22 μm membrane filter. Nutrient agar (NA) plates were inoculated with 100 μL of bacterial suspension adjusted to 10^8^ CFU/mL. Sterile paper disks were impregnated with the test solutions at a final concentration of 300 μg/disk and placed onto the inoculated agar surface. Commercial antibiotic disks—Ofloxacin (10 μg/disk), Sulbactam (30 μg) + Cefoperazone (75 μg) (total 105 μg/disk), or Netilmicin (30 μg/disk)—served as positive controls. Disks containing only the solvent were used as negative controls. Plates were incubated at 37°C for 24 h, after which the zones of inhibition were measured to assess antimicrobial activity. All tests were conducted at least in duplicate, in accordance with the guidelines of Wayne (2009).

### 2.18 Microwell Dilution Test

The microwell dilution test was performed to determine the Minimum Inhibitory Concentration (MIC) values of the test substances, which had demonstrated activity in the disk diffusion test. For this, 12‐h liquid cultures of the test bacteria were prepared and diluted to a 0.5 McFarland standard. The test substances and positive controls were prepared in two‐fold serial dilutions within the range of 500–7.8 μg/mL. In 96‐well microplates, 5 μL of bacterial culture and 95 μL of broth were added to each well. The highest concentration of the test substances (500 μg/mL) was added to the first well, with subsequent wells receiving the two‐fold dilutions. A negative control well contained no test substance, while the positive control wells contained antibiotics at varying concentrations (500–7.8 μg/mL). The plates were mixed at 300 rpm for 20 s and incubated at 37°C for 24 h. After incubation, microbial growth in each well was assessed by measuring absorbance at 600 nm. To verify the results, 5 μL was taken from the clear wells where microbial growth had been inhibited and inoculated onto appropriate solid media. The lowest concentration at which no growth occurred after incubation was considered to be the MIC, indicating the inhibitory potential of the substances. Each experiment was repeated at least twice, according to Wayne (2009).

### 2.19 Determination of Genotoxic and Antigenotoxic Activities

#### 2.19.1 Determination of Application Concentrations of Test Substances

The test substances were dissolved in dimethyl sulfoxide (DMSO) to prepare solutions for genotoxicity and antigenotoxicity testing. The upper limit for ideal application concentrations was established at 5 μg. To obtain reliable and meaningful results, each test substance was evaluated across at least five different concentrations, as recommended by Turhan et al. (2012).

#### 2.19.2 Genotoxic Activity Studies With Ames/*Salmonella* Test System

Genotoxicity was assessed using multiple test systems, including the Ames/Salmonella and *Allium cepa* assays. The Ames/*Salmonella* test was performed using the *Salmonella typhimurium* strains TA97a, TA98, TA100, TA1535, and TA1537 to identify potential gene mutations through single base substitution and insertion/deletion mutations. The following positive controls were used: 9‐aminoacridine (9‐AA, 50 μg/plate) for TA97a and TA1537, 4‐nitro‐o‐phenylenediamine (4‐NPD, 2.5 μg/plate) for TA98, and sodium azide (NaN₃, 5 μg/plate) for TA100 and TA1535. Dimethyl sulfoxide (DMSO, 100 μL/plate) served as the negative/vehicle control. In the mutagenicity assay, 100 μL of the test substance (2–10 mg/plate) and 100 μL of the test strain (*A*_540_: 0.1–0.2) were added to sodium phosphate buffer (pH: 7.4). The mixture was then combined with 2000 μL of molten agar containing limited L‐histidine, mixed well, and poured onto glucose minimal agar plates. After incubation at 37°C for 48 h, histidine‐independent colonies were counted. The results were analyzed using the plate incorporation method, and mutagenicity was confirmed if a two‐fold increase in colonies was observed in the test group compared to the negative/vehicle control group (Mortelmans and Zeiger 2000; Ozkan et al. 2014). All bacterial genotoxicity assays were repeated in triplicate, and the results were expressed as the mean of three samples with standard deviation.

### 2.20 *Allium cepa* Assay

For the *Allium cepa* assay, root precursors of individual bulbs were immersed in water and kept in the dark for 24 h at room temperature. Following this, ethyl methane sulfonate (EMS, 25 mM) was added to the test groups, EMS (25 mM) to the positive control group, and water to the negative control group. All groups were maintained under the same conditions for 48 h to allow for maximum cell division. Afterward, root tips were collected using sharp forceps, fixed in a solution of 45% acetic acid –1% HCl (9:1), and stained with 2% aceto‐orcein dye for 2 min. The lamellae were examined under a microscope to calculate the mitotic index (MI) and identify chromosomal abnormalities. The formula for MI is as follows: MI = (Number of dividing cells/Total number of cells) × 100. For the calculation of the mitotic index and chromosomal abnormalities, at least 5000 mitotic stem cells were observed at each concentration; the results were expressed as percentage (%) values and statistically analyzed with Fisher's exact test and linear trend (*p* < 0.05) (Feretti et al. 2007).

### 2.21 Investigation of Biosafety of Extracts/Active Substances With *Allium* Test

Onion tubers used in the study were procured from local markets. The experimental procedure began by peeling the outer shells of the tubers using sterile forceps. The tubers were then placed in pure water and left to root for 24 h at room temperature. After this period, only the tubers exhibiting healthy root growth were selected for further analysis. The following experimental groups were established:

- Negative Control (NK): Tubers developed in pure water
- Positive Control (PK): Tubers developed with ethyl methane sulfonate (EMS) treatment
- Test 1: Tubers developed with a 25 μg test substance treatment
- Test 2: Tubers developed with a 50 μg test substance treatment
- Test 3: Tubers developed with a 100 μg test substance treatment

The experimental groups were left to develop for an additional 24 h at room temperature. Following this incubation period, root samples were collected from each group and fixed using a solution of 45% acetic acid/1 N HCl (9:1) at 50°C for 6 min. Subsequently, 2 mm pieces were taken from the tips of the fixed roots using a sterile scalpel and transferred to slides, where they were stained with aceto‐orcein for 3 min. The preparations were covered with coverslips, which were slightly pressed to spread the cells. Cell examinations were conducted under a light microscope at 400× magnification, and at least 1000 cells were counted for each group. The cytogenetic status of the cells was recorded. The results were statistically analyzed using Fisher's exact test and linear trend analysis (Leme and Marin‐Morales 2009; Teixeira et al. 2003).

### 2.22 Statistical Analysis

All experiments were performed in triplicate. Statistical significance was assessed using the Kruskal–Wallis test. Data were analyzed using SPSS software (IBM SPSS Statistics 20, IBM Corporation, Armonk, NY, USA), with a significance level set at *p* = 0.05. The results are presented as the mean ± standard deviation (STD) of percent inhibition and IC50 values for the samples.

## 3 Results

### 3.1 Extraction

The flowering aerial parts of *S. macrantha* (50 g) and *S. hortensis* (30 g) were extracted with methanol by maceration for three days. Extracts were filtered and concentrated under reduced pressure below 40°C. Yield evaluation showed that the highest extraction efficiency was obtained from the aqueous extract of *S. hortensis* (42.15%), followed by the aqueous extract of *S. macrantha* (25.48%). In both species, aqueous extracts yielded more than methanolic ones (Table 1).

**TABLE 1:** The amounts of extracts obtained from *S. hortensis* and *S. macrantha* .

Species	Powdered (g)	Solvent	Extract amount (g)	Yield%
*S. macrantha*	50	MeOH	5.3916	10.7832
*S. hortensis*	30	MeOH	5.8433	19.477
*S. macrantha*	50	Water	12.7403	25.4806
*S. hortensis*	30	Water	12.6447	42.149

### 3.2 Essential Oil Extraction and Compositions

**FIGURE 1:**
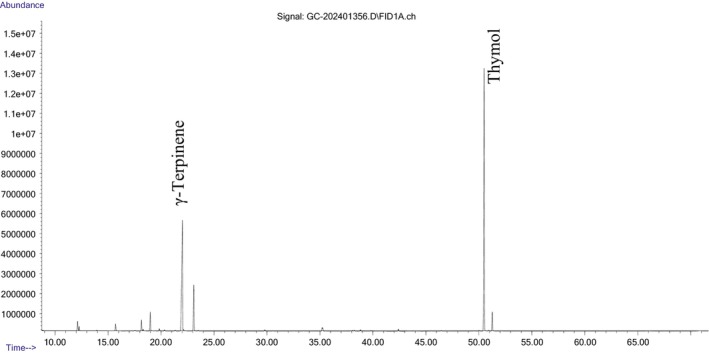
Chromatographic analysis of the essential oil of *S. hortensis* using GC–MS/MS.

**FIGURE 2:**
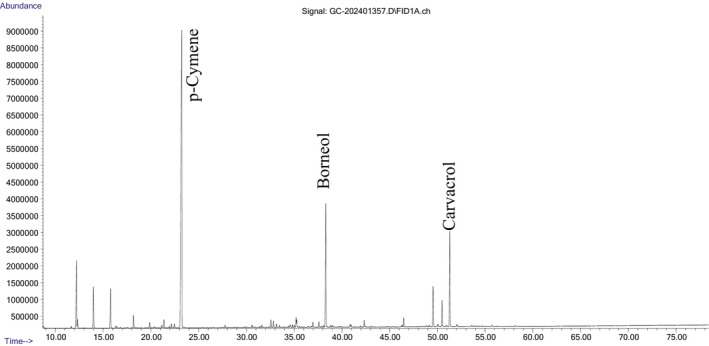
Chromatographic analysis of the essential oil of *S. macrantha* using GC–MS/MS.

The essential oils of *S. macrantha* and *S. hortensis* , obtained by hydrodistillation from their flowering aerial parts, were yellow in color, with yields of 0.2% and 0.1%, respectively. The chemical compositions of these oils were analyzed using both GC and GC–MS methods, and the results are presented in Table 2. Monoterpenes were found to be the predominant class of compounds in both essential oils, while sesquiterpenes and other constituents were present in smaller amounts. In *S. macrantha* , p‐cymene (45.8%) was the major compound, followed by borneol (11.3%), carvacrol (8.1%), α‐pinene (6.1%), camphene (3.8%), spathulenol (3.1%), and thymol (2.2%). In the essential oil of *S. hortensis* , thymol (45.1%) was identified as the dominant component, accompanied by γ‐terpinene (29.9%) and p‐cymene (8.2%). Other notable constituents included α‐terpinene (3.3%) and carvacrol (3.2%). Overall, the results confirmed the rich monoterpene content of both species' essential oils, with *S. macrantha* characterized by high p‐cymene levels, while *S. hortensis* was distinguished by its thymol content. Chromatographic analysis of the essential oils using GC–MS/MS was presented in Figures 1 and 2.

**TABLE 2:** Essential oil compositions of flowering aerial parts with flower of *S. macrantha* and *S. hortensis* .

KI^a^	RRI^b^	Compound	*S. hortensis* (%)	*S. macrantha* (%)	Identification method
998–1029^c^	1014	Tricyclene	—	0.2	MS
1008–1039^c^	1032	α‐Pinene	1.7	6.1	*t*_R_, MS
1012–1039^c^	1035	α‐Thujene	0.8	0.7	MS
1043–1086^c^	1076	Camphene	0.1	3.8	*t*_R_, MS
1085–1130^c^	1118	β‐Pinene	1.3	3.6	*t*_R_, MS
1098–1140^c^	1132	Sabinene	< 0.1	0.2	*t*_R_, MS
1140–1175^c^	1174	Myrcene	1.8	1.1	*t*_R_, MS
1148–1186^c^	1176	α‐Phellandrene	0.3	—	*t*_R_, MS
1154–1195^c^	1188	α‐Terpinene	3.3	< 0.1	*t*_R_, MS
1178–1219^c^	1203	Limonene	0.4	0.5	*t*_R_, MS
1186–1231^c^	1213	1,8‐Cineole	< 0.1	< 0.1	*t*_R_, MS
1188–1233^c^	1218	β‐Phellandrene	0.2	0.1	*t*_R_, MS
1196–1238^c^	1232	(*E*)‐2‐Hexenal	—	0.3	MS
1211–1251^c^	1246	(Z)‐β‐Ocimene	—	0.7	MS
1222–1266^c^	1255	γ‐Terpinene	29.9	0.1	*t*_R_, MS
1230–1280^c^	1265	3‐Octanone	—	0.4	MS
1232–1267^c^	1266	(*E*)‐β‐Ocimene	—	0.4	MS
1246–1291^c^	1280	p‐Cymene	8.2	45.8	*t*_R_, MS
1261–1300^c^	1290	Terpinolene	0.1	—	*t*_R_, MS
1331–1384^c^	1384	α‐Pinene oxide	—	< 0.1	MS
1372–1408^c^	1393	3‐Octanol	< 0.1	0.2	MS
1394–1457^d^	1452	α,p‐Dimethyl styrene	—	0.1	MS
1411–1465^c^	1452	1‐Octen‐3‐ol	0.2	—	MS
1425–1478^c^	1474	*cis*‐Sabinene hydrate	—	0.2	MS
1467^d^	1476	(*Z*)‐β‐Ocimene epoxide	—	0.1	MS
1462–1522^c^	1497	α‐Copaene	< 0.1	0.2	*t*_R_, MS
	1498	(*E*)‐β‐Ocimene epoxide	—	0.1	MS
1514–1530^d^	1528	α‐Bourbonene	—	< 0.1	MS
1481–1537^c^	1532	Camphor	—	0.6	*t*_R_, MS
1496–1546^c^	1535	β‐Bourbonene	—	0.6	MS
1507–1564^c^	1553	Linalool	—	0.3	*t*_R_, MS
1560–1590^c^	1568	*trans‐*α‐Bergamotene	—	0.2	MS
1545–1590^c^	1586	Pinocarvone	—	0.1	*t*_R_, MS
1547–1589^c^	1589	β‐Ylangene	—	0.1	MS
1563–1607^c^	1604	Thymol methyl ether	< 0.1	0.2	*t*_R_, MS
1564–1630^c^	1611	Terpinen‐4‐ol	0.5	0.6	*t*_R_, MS
1569–1632^c^	1612	β‐Caryophyllene	0.2	—	*t*_R_, MS
1576–1614^c^	1614	Carvacrol methyl ether	0.6	—	*t*_R_, MS
1583–1668^c^	1628	Aromadendrene	< 0.1	0.1	MS
1597–1648^c^	1648	Myrtenal	—	0.1	MS
1624–1668^c^	1661	Alloaromadendrene	—	< 0.1	MS
1643–1671^c^	1670	trans‐Pinocarveol	—	0.4	*t*_R_, MS
1643–1671^c^	1683	trans‐Verbenol	—	0.5	*t*_R_, MS
1637–1689^c^	1687	α‐Humulene	< 0.1	—	*t*_R_, MS
1659–1724^c^	1706	α‐Terpineol	0.1	0.1	*t*_R_, MS
1656–1736^d^	1708	Ledene	< 0.1	—	MS
1653–1728^c^	1719	Borneol	0.1	11.3	*t*_R_, MS
1698–1748^c^	1741	β‐Bisabolene	0.2	0.1	*t*_R_, MS
1699–1751^c^	1751	Carvone	—	0.1	*t*_R_, MS
1692–1757^c^	1755	Bicyclogermacrene	—	—	MS
1722–1774^c^	1773	δ‐Cadinene	0.1	0.1	MS
1735–1782^c^	1776	γ‐Cadinene	< 0.1	< 0.1	MS
1747–1805^c^	1802	Cumin aldehyde	—	0.2	*t*_R_, MS
1743–1808^c^	1804	Myrtenol	—	0.3	MS
1805–1850^c^	1845	trans‐Carveol	—	0.1	*t*_R_, MS
1813–1865^c^	1864	p‐Cymen‐8‐ol	—	0.5	*t*_R_, MS
1783–1945^c^	1867	Thymyl acetate	0.3	0.1	*t*_R_, MS
1818–1882^c^	1882	cis‐Carveol	—	< 0.1	*t*_R_, MS
1868–1890^c^	1890	Carvacryl acetate	< 0.1	0.1	*t*_R_, MS
1936–2023^c^	2008	Caryophyllene oxide	0.1	0.7	*t*_R_, MS
2068–2115^d^	2113	Cumin alcohol	—	0.1	*t*_R_, MS
2074–2150^c^	2144	Spathulenol	0.1	3.1	MS
	2181	Isothymol	0.1	—	*t*_R_, MS
2100–2205^c^	2198	Thymol	45.1	2.2	*t*_R_, MS
	2221	Isocarvacrol	0.1	—	*t*_R_, MS
2140–2246^c^	2239	Carvacrol	3.2	8.1	*t*_R_, MS
2510–2633^c^	2622	Phytol	—	—	MS
		Total	98.3	95.9	

^a^ Relative retention indices reported in the literature.

^b^ Relative retention indices (RRI) experimentally determined against *n*‐alkanes; % calculated from FID data; IM, identification method; MS, identified on the basis of computer matching of the mass spectra with those of the Wiley and MassFinder libraries and comparison with literature data; *t*R, identification based on the retention times of genuine compounds on the HP Innowax column.

^c^ Babushok et al. (2011).

^d^ Pubchem (https://pubchem.ncbi.nlm.nih.gov/compound/ (accessed on 29 November 2024)).

### 3.3 Qualitative and Quantitative Analysis of Secondary Metabolites

**FIGURE 3:**
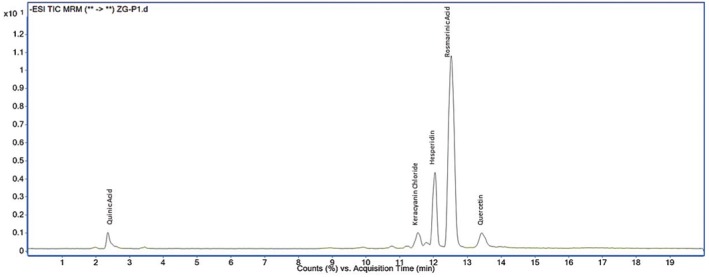
Chromatographic analysis of the water extract using LC–MS/MS.

**FIGURE 4:**
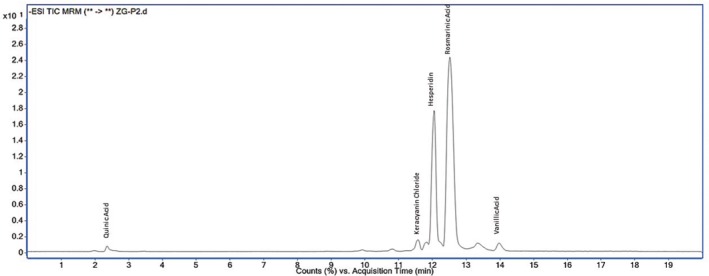
Chromatographic analysis of the methanol extract using LC–MS/MS.

The LC–MS/MS profiles of the most active methanol and water extracts of *S. macrantha* flowering aerial parts are presented in Table 3. A total of 35 phenolic compounds were identified across both extracts, including key constituents such as rosmarinic acid, hesperidin, chlorogenic acid, catechin, and quercetin. Quantitative analysis showed that rosmarinic acid and hesperidin were the dominant phenolics in both extracts. In the water extract, rosmarinic acid was found at 50,687.02 ng/mL and hesperidin at 6089.64 ng/mL, with 11 compounds identified overall. The methanol extract contained 130,792.51 ng/mL of rosmarinic acid and 25,749.17 ng/mL of hesperidin, along with 15 phenolic compounds. These findings highlight the rich phenolic content of *S. macrantha* , particularly in the methanol extract, supporting its potential biological activity. Chromatographic analysis of the water and methanol extracts using LC–MS/MS was presented in Figures 3 and 4.

**TABLE 3:** LC–MS/MS qualitative and quantitative analysis results of *S. macrantha* water and methanol extracts.

Sayı	Compound	Water extract Concentration (ng/mL)	Methanol extract Concentration (ng/mL)
1.	Quinic acid	4295.5525	3282.4918
2.	Keracyanin chloride	2440.6531	6071.3863
3.	Cyanidin‐3‐O‐glucoside	157.0393	488.2374
4.	Chlorogenic acid	80.4062	205.4768
5.	Vanillic acid	0.0000	4240.6607
6.	Syringic acid	0.0000	21.9875
7.	Naringenin	0.0000	85.1219
8.	Naringin	0.0000	117.3606
9.	Hesperidin	6089.6389	25,749.1685
10.	Ferulic acid	0.0000	1266.5583
11.	Peonidin‐3‐O‐glucoside	2.4397	0.0000
12.	Caffeic acid	72.4307	0.0000
13.	Luteolin	0.0000	78.6366
14.	Rosmarinic acid	50,687.0234	130,792.5115
15.	Vanillin	43.7733	44.0385
16.	Resveratrol	2.4075	2.1134
17.	Quercetin	286.2336	124.7067

### 3.4 Total Phenolic Content Quantification

Total phenolic contents (TPC) of water and methanol extracts from *S. macrantha* and *S. hortensis* were calculated using gallic acid as a standard and are presented in Table 4. Overall, water extracts showed the highest TPC, followed by methanol extracts, while essential oils contained the lowest amounts due to their non‐polar nature. The water extract of *S. macrantha* had the highest TPC (131.305 μg GAE/mg), approximately 4.5 times greater than that of *S. hortensis* (29.305 μg GAE/mg). The methanol extract of *S. macrantha* also exhibited higher phenolic content (74.944 μg GAE/mg) compared to *S. hortensis* (35.222 μg GAE/mg). Interestingly, in essential oils, the trend reversed: *S. hortensis* oil contained significantly more phenolics (60.722 μg GAE/mg) than *S. macrantha* oil (12.444 μg GAE/mg). These results emphasize the impact of extraction solvent on phenolic yield and suggest that the aqueous and methanol extracts of *S. macrantha* may possess superior antioxidant potential, while the relatively high phenolic content in *S. hortensis* essential oil may contribute to its bioactivity through volatile phenolics.

**TABLE 4:** Total phenolic content of *S. hortensis* and *S. macrantha* extracts.

Samples	Total phenolic content (μg GAE/mg extract ± standard deviation)
*S. macrantha*	
Water	131.305 ± 0.0017
MeOH	74.944 ± 0.0067
Essential oil	12.444 ± 0.0011
*S. hortensis*	
Water	29.305 ± 0.0004
MeOH	35.222 ± 0.0014
Essential oil	60.722 ± 0.0005

*Note:* Results are expressed as mean ± standard deviation (*n* = 3). Values are expressed as mean ± SD (*n* = 3). Significant differences were determined using *t*‐test; *p* < 0.05 indicates statistical significance.

### 3.5 Total Flavonoid Content Quantification

Total flavonoid contents (TFC) of water and methanol extracts from *S. macrantha* and *S. hortensis* , calculated using gallic acid as a standard, are presented in Table 5. Flavonoids, known for their antioxidant and anti‐inflammatory properties, varied depending on both species and solvent type. The water extract of *S. macrantha* showed the highest TFC (783.333 μg GAE/mg), approximately 4.5 times greater than that of *S. hortensis* (171.333 μg GAE/mg). Likewise, the methanol extract of *S. macrantha* had a higher flavonoid content (445.166 μg GAE/mg) than *S. hortensis* (206.833 μg GAE/mg). Interestingly, while *S. macrantha* yielded more flavonoids in water, *S. hortensis* exhibited a slightly higher TFC in its methanol extract than in its water extract, suggesting species‐specific differences in flavonoid solubility and composition. These findings indicate that *S. macrantha* is significantly richer in flavonoids, particularly in its aqueous extract, supporting its potential for strong antioxidant activity. The results also highlight the importance of solvent selection for efficient extraction of flavonoid‐rich fractions in phytochemical and pharmacological studies.

**TABLE 5:** Total flavonoid content of *S. hortensis* and *S. macrantha* extracts.

Samples	Total flavonoid content (μg GAE/mg extract ± standard deviation)
*S. macrantha*	
Water	783.333 ± 0.0017
MeOH	445.166 ± 0.0067
*S. hortensis*	
Water	171.333 ± 0.0004
MeOH	206.833 ± 0.0014

*Note:* Results are expressed as mean ± standard deviation (*n* = 3). Values are expressed as mean ± SD (*n* = 3). Significant differences were determined using *t*‐test; *p* < 0.05 indicates statistical significance.

### 3.6 Total Tannin Content Quantification

Total tannin content (TTC) of water and methanol extracts from *Satureja macrantha* and *S. hortensis* , calculated using gallic acid as a standard, is presented in Table 6. Tannins, known for their antioxidant and antimicrobial properties, were more abundant in water extracts, reflecting their higher solubility in polar solvents. Among the tested samples, *S. macrantha* water extract had the highest TTC (251.333 μg TAE/mg), approximately 38 times greater than that of *S. hortensis* (6.533 μg TAE/mg). The methanol extract of *S. macrantha* also exhibited significantly higher tannin content (116.066 μg TAE/mg) compared to *S. hortensis* (20.733 μg TAE/mg). These results demonstrate that *S. macrantha* is a substantially richer source of tannins than *S. hortensis* , particularly in aqueous extracts, suggesting stronger antioxidant and antimicrobial potential. In contrast, the low tannin content in *S. hortensis* indicates that its biological effects may be attributed to other classes of phytochemicals. Overall, the findings emphasize the importance of solvent selection in optimizing tannin extraction and support the potential use of *S. macrantha* as a natural source of bioactive tannins in pharmaceutical, cosmetic, or food‐related applications.

**TABLE 6:** Total tannin content of *S. hortensis* and *S. macrantha* extracts.

Samples	Total tannin content (μg TAE/mg extract ± standard deviation)
*S. macrantha*	
Water	251.333 ± 0.0017
MeOH	116.066 ± 0.0067
*S. hortensis*	
Water	6.533 ± 0.0004
MeOH	20.733 ± 0.0014

*Note:* Results are expressed as mean ± standard deviation (*n* = 3). Values are expressed as mean ± SD (*n* = 3). Significant differences were determined using *t*‐test; *p* < 0.05 indicates statistical significance.

### 3.7 ABTS˙ ^+^ and DPPH˙ Cation Radical Scavenging Capacity

The antioxidant capacities of extracts and essential oils from *Satureja macrantha* and *S. hortensis* at 50 μg/mL concentration are summarized in Table 7, based on ABTS˙ ^+^ and DPPH˙ scavenging assays. Standard antioxidants α‐tocopherol and Trolox were used as references. In the ABTS˙ ^+^ assay, Trolox showed the highest inhibition (65.7%), followed by the water extract of *S. macrantha* (52.1%), which also exceeded the activity of α‐tocopherol. The methanol extract of *S. hortensis* showed moderate activity (17.6%), while the essential oils exhibited low inhibition ( *S. macrantha* : 9.9%, *S. hortensis* : 10.7%). In the DPPH˙ assay, Trolox again demonstrated the strongest activity (93.9%), followed by α‐tocopherol (68.1%). Among plant samples, the water extract of *S. macrantha* showed the highest inhibition (28.8%), whereas the water extract of *S. hortensis* had the lowest (4.4%). Essential oils of both species exhibited minimal DPPH˙ scavenging (10.1% and 4.3%, respectively). These results are consistent with total phenolic content data and highlight the strong antioxidant potential of *S. macrantha* , particularly in its aqueous extract, while essential oils showed limited activity in both assays.

**TABLE 7:** ABTS^+^ and DPPH˙ scavenging capacity results of *S. hortensis* and *S. macrantha* extracts.

Samples	ABTS^+^ scavenging capacity (50 μg/mL % inhibition ± standard deviation)	DPPH˙ scavenging capacity (50 μg/mL % inhibition ± standard deviation)
α‐Tocopherol	11.162 ± 0.0494	68.063 ± 0.0241
Trolox	65.697 ± 0.0123	93.891 ± 0.0002
*S. macrantha*		
Water	52.111 ± 0.0438	28.762 ± 0.0184
MeOH	18.576 ± 0.0136	13.748 ± 0.0434
Essential oil	9.903 ± 0.0231	10.064 ± 0.0038
*S. hortensis*		
Water	9.233 ± 0.0195	4.396 ± 0.0245
MeOH	17.611 ± 0.0146	—
Essential oil	10.696 ± 0.0012	4.348 ± 0.0138

*Note:* Results are expressed as mean ± standard deviation (*n* = 3). Values are expressed as mean ± SD (*n* = 3). Significant differences were determined using *t*‐test; *p* < 0.05 indicates statistical significance.

### 3.8 α‐Glucosidase and α‐Amylase Enzyme Inhibition Determination

The α‐glucosidase inhibition percentages of *S. hortensis* and *S. macrantha* extracts and essential oils at a concentration of 1000 μg/mL are presented in Table 8. For α‐glucosidase inhibition, *S. macrantha* water extract showed the highest inhibition at 49.4%, followed by its methanol extract at 39.4%. The essential oil of *S. macrantha* showed no detectable inhibition (N.D.), and no α‐glucosidase inhibition was observed in the extracts or essential oils of *S. hortensis* . Acarbose, the reference standard, inhibited α‐glucosidase by 34.98%. For α‐amylase inhibition, acarbose demonstrated strong inhibition at 64.5%. However, none of the *Satureja* extracts or essential oils showed significant α‐amylase inhibition, as all values are marked as N.D. except for the *S. hortensis* essential oil, which exhibited a modest inhibition of 41.33%.

**TABLE 8:** α‐Glucosidase and α‐amylase inhibition (%) values of extracts and acarbose at 1000 μg/mL concentration.

Samples	α‐Glucosidase 1000 μg/mL % inhibition ± standard deviation	α‐amylase %5000 μg/mL % inhibition ± standard deviation
Acarbose	34.98 ± 2.08	64.50 ± 0.19
*S. macrantha*		
Water	49.36 ± 0.53	ND
MeOH	39.43 ± 6.08	ND
Essential oil	ND	ND
*S. hortensis*		
Water	ND	ND
MeOH	ND	ND
Essential oil	41.33 ± 4.87	ND

*Note:* Results are expressed as mean ± standard deviation (*n* = 3). Values are expressed as mean ± SD (*n* = 3). Significant differences were determined using *t*‐test; *p* < 0.05 indicates statistical significance.

Abbreviation: ND, not determined.

In conclusion, *S. macrantha* water extract demonstrated notable α‐glucosidase inhibition, while *S. hortensis* essential oil showed a moderate effect. Both species' extracts and essential oils showed little to no α‐amylase inhibition compared to the positive control, acarbose.

### 3.9 Acetylcholinesterase (AChE) and Butyrylcholinesterase (BChE) Inhibition Assays

The acetylcholinesterase (AChE) and butyrylcholinesterase (BChE) inhibitory activities of *Satureja hortensis* and *Satureja macrantha* extracts and essential oils are summarized in Table 9. Donepezil, used as a positive control, exhibited 100% inhibition in both assays. For AChE inhibition, *S. macrantha* showed modest activity: the methanol extract inhibited 12.72%, the water extract 9.80%, and the essential oil 7.84%. In contrast, *S. hortensis* demonstrated slightly higher inhibition, with the methanol extract (16.05%) and essential oil (14.41%) being the most effective, while the water extract showed minimal activity (6.56%). In the BChE inhibition assay, *S. macrantha* methanol extract showed moderate activity (22.33%), whereas the water extract (9.86%) and essential oil (10.45%) were less effective. *S. hortensis* displayed more pronounced BChE inhibition, with the essential oil achieving 35.68% and the methanol extract 28.01%; no inhibition was detected in the water extract (N.D.). In conclusion, while both species exhibited weak AChE inhibition relative to donepezil, *S. hortensis* —particularly its essential oil and methanol extract—showed notable BChE inhibitory activity, suggesting potential relevance in neuroprotective applications.

**TABLE 9:** AChE and BChE inhibition (%) values of samples and donepezil at 100 μg/mL concentration.

Samples	AChE (100 μg/mL % inhibition ± standard deviation)	BChE (1000 μg/mL % inhibition ± standard deviation)
Donepezil	100 ± 1.41	100 ± 0.80
*S. macrantha*		
Water	9.80 ± 3.41	9.86 ± 5.73
MeOH	12.72 ± 2.94	22.33 ± 4.99
Essential oil	7.84 ± 3.31	10.45 ± 3.56
*S. hortensis*		
Water	6.56 ± 1.08	ND
MeOH	16.05 ± 2.42	28.01 ± 2.87
Essential oil	14.41 ± 3.66	35.68 ± 3.61

*Note:* Results are expressed as mean ± standard deviation (*n* = 3). Values are expressed as mean ± SD (*n* = 3). Significant differences were determined using *t*‐test; *p* < 0.05 indicates statistical significance.

Abbreviation: ND, not determined.

### 3.10 Antimicrobial Activity

The antimicrobial activity results of *S. macrantha* and *S. hortensis* extracts and essential oils, along with the tested microorganisms, are presented in Table 10. Minimum inhibitory concentration (MIC) values showed that the essential oils exhibited stronger antimicrobial effects compared to the extracts. Specifically, *S. hortensis* essential oil demonstrated the lowest MIC value (50 μg/mL), followed by *S. macrantha* essential oil (125 μg/mL). Both water and methanol extracts of the two species showed higher MIC values (500 μg/mL), indicating weaker activity. In the disc diffusion assay, *S. macrantha* methanol extract was inactive, while its water extract showed limited activity against *Bacillus cereus* (6 mm inhibition zone). The essential oil of *S. macrantha* exhibited moderate activity, with inhibition zones ranging from 6 to 8 mm against several strains, including *S. aureus* , *S. mutans* , *E. faecalis* , and *B. cereus* . For *S. hortensis* , the essential oil displayed the strongest antimicrobial effects, with inhibition zones of 10 mm against *S. aureus* , *A. baumannii* , *E. coli* , *S. mutans* , and *B. cereus* . It also showed 8 mm inhibition against *E. faecium* and *S. salivarius* , and 7 mm against *E. faecalis* . The methanol extract of *S. hortensis* exhibited weak activity, only against *S. mutans* (6 mm), with no effect on other strains. These results suggest that *S. hortensis* essential oil has the most potent and broad‐spectrum antimicrobial activity, while the extracts, particularly methanol, showed limited or no effects. The findings support the potential of essential oils, especially from *S. hortensis* , as promising natural antimicrobial agents.

**TABLE 10:** Antimicrobial activity results of *S. macrantha* and *S. hortensis* extracts and essential oils.

Disk diffusion test results (mm)									
Samples	A	B	C	D	E	F	G	H	J
*S. macrantha*									
MeOH	—	—	—	—	—	—	—	—	—
Water	—	—	—	—	—	—	—	—	6 mm (+)
Essential oil	8 mm (++)	—	—	—	—	—	6 mm (+)	8 mm (++)	7 mm (+)
*S. hortensis*									
MeOH	—	—	—	—	—	—	6 mm (+)	—	—
Water	—	—	—	—	—	—	—	—	—
Essential oil	10 mm (+++)	10 mm (+++)	8 mm (++)	10 mm (+++)	8 mm (++)	—	10 mm (+++)	7 mm (++)	10 mm (+++)
Positive control	22	20	18	24	21	19	23	20	25

*Note:* A, *Methicillin‐resistant Staphylococcus aureus* ATCC 67106; B, *Acinetobacter baumannii* ATCC BA1609; C, *Enterococcus faecium* ATCC 700211; D, *Escherichia coli* ATCC BAA‐2523; E, *Streptococcus salivarius* ATCC 13419; F, *Pseudomonas aeruginosa* ATCC 9070; G, *Streptococcus mutans* ATCC 35668; H, *Enterococcus faecalis* ATCC 49452; J, *Bacillus cereus* ATCC 14579.

### 3.11 Determination of Genotoxic and Antigenotoxic Activities

**FIGURE 5:**
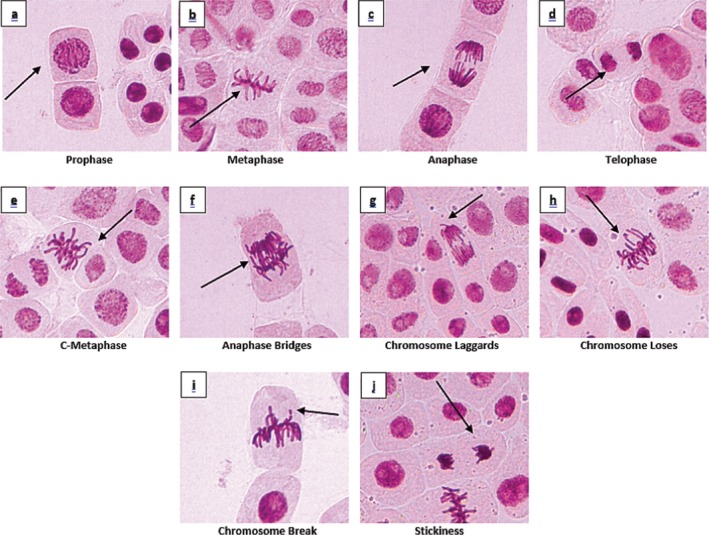
Typical stages of mitosis (a–d) and chromosomal aberrations (e–j) induced by EMS in mitotic *A. cepa* root cells.

The genotoxic safety of *S. hortensis* and *S. macrantha* extracts and essential oils was evaluated using Ames/*Salmonella* and *Allium cepa* test systems. The Ames test employed five *Salmonella typhimurium* strains (TA97a, TA98, TA100, TA1535, TA1537) with concentrations ranging from 20 to 100 μg/petri. No significant increase in revertant colonies was observed compared to the negative control, indicating no mutagenic effect (Table 11). All tests were conducted in accordance with ICH and FDA guidelines and repeated in triplicate. The *Allium cepa* assay assessed cytogenotoxicity at concentrations between 0.1 and 0.5 mg, with distilled water and ethyl methanesulfonate (25 mM) serving as negative and positive controls, respectively. Water and methanol extracts of both species showed no significant cytogenetic toxicity, maintaining a normal mitotic index and chromosomal structure. However, the essential oils of both species caused dose‐dependent reductions in mitotic index and increased chromosomal aberrations, indicating potential genotoxic effects at higher concentrations (Table 12 and Figure 5). Additionally, although slight increases in colony numbers were observed with higher extract doses in the Ames test—especially for *S. macrantha* —these remained within the acceptable range and were not statistically significant. Notably, *S. hortensis* essential oil showed cytotoxicity at higher doses, highlighting the importance of dose optimization for potential therapeutic use. These findings suggest that while methanol and water extracts are genotoxically safe, the essential oils may present cytogenetic risks at high concentrations, warranting further investigation before pharmaceutical application.

**TABLE 11:** Genotoxic activity results of *Satureja* extracts and essential oils.

Test groups	Reverse mutant colony numbers of *S. typhimurium* test strains (mean ± SD)				
	*S. typhimurium* TA1535	*S. typhimurium* TA100	*S. typhimurium* TA1537	*S. typhimurium* TA97a	*S. typhimurium* TA98
*S. macrantha* —MeOH (μg/petri)					
100	34.0 ± 3.29	42.8 ± 3.60	22.5 ± 2.59	46.7 ± 1.63	58.5 ± 2.17
80	34.3 ± 1.86	44.2 ± 3.43	21.0 ± 2.83	48.2 ± 4.45	58.3 ± 3.67
60	32.7 ± 2.80	41.7 ± 3.08	20.7 ± 1.75	49.3 ± 2.73	58.3 ± 4.08
40	33.3 ± 3.72	41.0 ± 3.52	21.3 ± 2.66	49.0 ± 2.10	58.2 ± 3.92
20	32.5 ± 2.07	43.3 ± 4.32	21.7 ± 2.25	49.7 ± 4.80	57.7 ± 2.66
*S. macrantha* —water (μg/petri)					
100	35.8 ± 2.32	44.8 ± 4.45	21.2 ± 2.64	49.0 ± 3.74	62.2 ± 1.83
80	34.2 ± 2.86	44.7 ± 2.66	21.7 ± 2.66	49.0 ± 3.10	59.0 ± 3.74
60	33.2 ± 3.60	43.0 ± 4.34	21.0 ± 2.83	47.8 ± 4.45	58.7 ± 2.80
40	34.5 ± 3.45	43.2 ± 3.43	21.0 ± 2.90	47.0 ± 3.58	59.3 ± 4.03
20	33.3 ± 2.66	42.0 ± 2.53	21.5 ± 2.35	51.7 ± 3.44	58.8 ± 4.36
*S. macrantha* —essential oil (μg/petri)					
100	34.7 ± 3.27	43.8 ± 3.31	22.5 ± 1.87	48.5 ± 4.64	Cytotoxic
80	35.5 ± 3.62	43.8 ± 3.66	20.5 ± 2.59	50.8 ± 4.36	62.7 ± 4.46
60	35.3 ± 3.08	45.3 ± 3.61	20.3 ± 2.34	49.2 ± 3.97	60.2 ± 3.71
40	33.5 ± 2.74	43.7 ± 3.61	20.5 ± 3.02	50.5 ± 3.73	62.5 ± 4.55
20	34.5 ± 4.14	44.7 ± 4.13	21.5 ± 3.56	50.3 ± 1.03	59.8 ± 1.94
*S. hortensis* —MeOH (μg/petri)					
100	36.8 ± 1.60	43.7 ± 3.39	20.2 ± 2.48	48.8 ± 4.71	61.3 ± 3.08
80	32.7 ± 3.39	42.8 ± 5.04	19.3 ± 1.51	51.3 ± 3.61	58.5 ± 4.59
60	32.2 ± 2.48	45.3 ± 2.66	19.7 ± 1.37	45.8 ± 2.79	61.8 ± 2.79
40	34.3 ± 2.66	46.5 ± 1.52	21.2 ± 3.19	49.0 ± 3.69	59.0 ± 3.35
20	32.5 ± 4.04	43.3 ± 3.14	19.2 ± 2.32	48.2 ± 3.66	60.3 ± 3.20
*S. hortensis* —water (μg/petri)					
100	31.0 ± 2.00	44.3 ± 3.72	21.2 ± 2.56	48.0 ± 3.41	59.5 ± 4.85
80	34.2 ± 3.60	44.0 ± 4.38	20.8 ± 2.64	44.7 ± 1.75	59.5 ± 3.99
60	33.3 ± 3.88	43.2 ± 3.25	21.2 ± 2.48	48.5 ± 1.97	59.2 ± 3.87
40	35.3 ± 2.07	43.3 ± 3.08	20.8 ± 2.56	48.2 ± 5.04	61.5 ± 3.73
20	34.7 ± 3.72	44.5 ± 2.81	21.5 ± 2.59	46.8 ± 2.93	60.2 ± 3.19
*S. hortensis* —essential oil (μg/petri)					
100	Cytotoxic	Cytotoxic	Cytotoxic	Cytotoxic	Cytotoxic
80	Cytotoxic	Cytotoxic	Cytotoxic	Cytotoxic	Cytotoxic
60	Cytotoxic	Cytotoxic	Cytotoxic	Cytotoxic	Cytotoxic
40	36.7 ± 2.25	44.0 ± 3.63	21.7 ± 2.34	48.8 ± 4.07	61.7 ± 2.73
20	32.3 ± 2.88	45.5 ± 3.67	20.7 ± 1.97	48.2 ± 3.54	60.2 ± 3.54
Positive controls					
NaN_3_ (5 μg)	523.7 ± 14.14	518.7 ± 11.71	347.8 ± 7.94	496.0 ± 15.54	613.0 ± 14.63
9‐AA (50 μg)					
4‐NPD (2.5 μg)					
Negative control					
DMSO (100 μL)	31.8 ± 3.60	45.5 ± 3.45	23.2 ± 1.83	50.8 ± 3.25	60.8 ± 4.07

*Note:* Results are expressed as mean ± standard deviation (*n* = 3). Values are expressed as mean ± SD (*n* = 3). Significant differences were determined sing *t*‐test; *p* < 0.05 indicates statistical significance.

**TABLE 12:** Mitotic index and chromosome abnormalities in *A. cepa* roots exposed to *Satureja* extracts and essential oils.

Test groups (mg/mL)	Mitotic index (%)	Chromosome aberrations (%)							Statistical testing	
		AB	CB	CLag	CLos	CM	ST	T	Fisher's exact test	Linear trend
*S. macrantha* —MeOH										
0.1	21.0	0.1	0.1	0.3	0.1	0.0	0.1	0.7	NS	NS
0.2	21.2	0.1	0.0	0.3	0.0	0.0	0.1	0.5	NS	
0.3	21.2	0.1	0.1	0.2	0.0	0.1	0.0	0.5	NS	
0.4	20.9	0.3	0.1	0.1	0.0	0.0	0.0	0.5	NS	
0.5	21.3	0.2	0.0	0.2	0.1	0.0	0.1	0.6	NS	
*S. macrantha* —water										
0.1	21.2	0.1	0.1	0.1	0.1	0.0	0.2	0.6	NS	NS
0.2	20.5	0.2	0.1	0.1	0.0	0.0	0.1	0.5	NS	
0.3	20.9	0.2	0.2	0.2	0.0	0.0	0.1	0.7	NS	
0.4	21.5	0.2	0.1	0.2	0.0	0.0	0.1	0.6	NS	
0.5	20.1	0.1	0.1	0.3	0.0	0.0	0.2	0.8	NS	
*S. macrantha* —essential oil										
0.1	21.1	0.3	0.2	0.2	0.0	0.0	0.1	0.8	NS	S
0.2	20.6	0.2	0.1	0.2	0.1	0.1	0.1	0.8	NS	
0.3	16.8	0.3	0.3	0.2	0.2	0.3	0.2	1.5	S	
0.4	15.3	0.9	0.4	0.4	0.2	0.1	0.4	2.4	S	
0.5	14.9	1.3	0.4	0.4	0.2	0.3	0.4	3.0	S	
*S. hortensis* —MeOH										
0.1	21.2	0.1	0.1	0.2	0.1	0.1	0.1	0.7	NS	NS
0.2	21.2	0.1	0.1	0.1	0.1	0.0	0.0	0.4	NS	
0.3	21.2	0.2	0.1	0.1	0.1	0.0	0.2	0.7	NS	
0.4	19.7	0.1	0.2	0.1	0.0	0.0	0.1	0.5	NS	
0.5	21.7	0.1	0.1	0.1	0.0	0.0	0.2	0.5	NS	
*S. hortensis* —water										
0.1	20.0	0.2	0.2	0.2	0.0	0.0	0.1	0.7	NS	NS
0.2	20.5	0.2	0.1	0.2	0.0	0.0	0.1	0.6	NS	
0.3	22.2	0.2	0.2	0.2	0.1	0.0	0.0	0.7	NS	
0.4	20.4	0.2	0.1	0.1	0.0	0.0	0.2	0.6	NS	
0.5	21.1	0.1	0.1	0.3	0.1	0.0	0.0	0.6	NS	
*S. hortensis* —essential oil										
0.1	17.1	1.1	0.1	0.1	0.1	0.1	0.1	1.6	S	S
0.2	16.5	1.4	0.1	0.2	0.3	0.1	0.2	2.3	S	
0.3	14.2	1.7	0.2	0.4	0.2	0.3	0.7	3.5	S	
0.4	13.5	2.0	0.2	0.7	0.2	0.3	0.9	4.3	S	
0.5	11.1	2.2	0.4	1.2	0.3	0.4	1.3	5.8	S	
Control groups										
Negative C.: ddH_2_O	21.3	0.1	0.2	0.2	0.1	0.0	0.1	0.7		
Positive C.: EMS (25 mM)	13.4	2.1	0.4	1.0	0.6	0.5	1.1	5.7		

*Note:* Values are expressed as mean ± SD (*n* = 3). Significant differences were determined using *t*‐test; *p* < 0.05 indicates statistical significance.

Abbreviations: AB, anaphase bridge; CB, chromosome breakage; CLag, chromosome lag; CLos, chromosome loss; CM, C‐meta phase; NS, not statistically significant compared with negative control (*p* < 0.05); S, statistically significant; ST, stickiness; T, total chromosome aberration rate.

## 4 Discussion

This study demonstrates that *S. macrantha* and *S. hortensis* differ significantly in their phytochemical compositions and biological activities. The main finding is that *S. macrantha* water extract exhibited the strongest antioxidant potential, closely associated with its high phenolic, flavonoid, and tannin content. In contrast, *S. hortensis* essential oil showed the most potent antimicrobial activity, particularly against clinically relevant bacterial strains. Genotoxicity tests confirmed the safety of the methanol and water extracts from both species, although essential oils exhibited cytotoxic effects at higher concentrations. These results underline the importance of species selection, extraction method, and chemical profile when evaluating the therapeutic potential of medicinal plants. The data support the potential application of *S. macrantha* in antioxidant formulations and *S. hortensis* essential oil as a natural antimicrobial agent.

### 4.1 Essential Oil Extraction and Compositions

Numerous studies have reported considerable variation in the essential oil compositions of different *Satureja* species, largely influenced by geographical origin and environmental conditions. For instance, Sefidkon and Jamzad (2005) identified carvacrol (30.9%), thymol (26.5%), γ‐terpinene (14.9%), and p‐cymene (10.3%) as major constituents in *S. mutica* , while *S. macrantha* was dominated by p‐cymene (25.8%), limonene (16.3%), and thymol (8.1%). Similarly, the oil of *S. intermedia* contained high levels of thymol (32.3%), γ‐terpinene (29.3%), and p‐cymene (14.7%). In our study, *S. hortensis* was characterized by 29.9% γ‐terpinene, 8.2% p‐cymene, 3.2% carvacrol, and 0.4% limonene, whereas *S. macrantha* contained 45.8% p‐cymene, 8.1% carvacrol, 2.2% thymol, 0.5% limonene, and 0.1% γ‐terpinene. Altun and Goren (2007) analyzed *S. cuneifolia* from Çanakkale, identifying thymol (35.8%), p‐cymene (18.3%), and γ‐terpinene (14.2%) as major components, along with p‐cymene‐2,5‐dione and β‐myrcene—compounds that were not detected in our *S. hortensis* or *S. macrantha* samples. Instead, our samples contained notable levels of α‐terpinene, carvacrol, and p‐cymene. Cavar et al. (2008) reported thymol (31.7%) and geraniol (22.3%) as main components of *S. montana* , while *S. subspicata* was dominated by thymol (28.6%) and spathulenol (37.6%). Geraniol was absent in our samples, but *S. macrantha* contained 3.1% spathulenol, and *S. hortensis* contained only 0.1%. Skočibušić et al. (2006) found high levels of carvacrol (16.76%), α‐pinene (13.58%), and p‐cymene (10.76%) in *S. subspicata*, along with several minor compounds. In contrast, *S. macrantha* in our study showed higher p‐cymene (45.8%) and borneol (11.3%) levels, and trace amounts of many other shared constituents. *S. hortensis* was rich in thymol (45.1%), followed by γ‐terpinene (29.9%) and p‐cymene (8.2%), with several minor terpenes also present. Kızıl (2009) examined *S. hortensis* from Diyarbakır and Kahramanmaraş, reporting carvacrol and thymol as dominant, with considerable variation between regions. Our *S. macrantha* samples from Iran contained notably lower levels of carvacrol (8.1%) and thymol (2.2%), but higher p‐cymene content (45.8%). These comparisons emphasize that the essential oil profiles of *Satureja* species can differ greatly, not only between species but also within the same species across different regions. Such variability highlights the need for localized phytochemical studies, particularly when evaluating these plants for therapeutic or industrial applications.

5

### 5.1 Qualitative and Quantitative Analysis of Secondary Metabolites

Rosmarinic acid is a naturally occurring phenolic ester formed from caffeic acid and 3,4‐dihydroxyphenyllactic acid, and is commonly found in members of the Boraginaceae family and the Nepetoideae subfamily within the Lamiaceae (Hitl et al. 2021; Petersen and Simmonds 2003). This compound is a key contributor to the medicinal value of various herbs and spices due to its potent antioxidant, anti‐inflammatory, antibacterial, and antiviral activities (Petersen and Simmonds 2003). It is especially abundant in species such as *Rosmarinus officinalis* , *Perilla* spp., and *Salvia officinalis* , and has been associated with therapeutic benefits in conditions like asthma, allergic rhinitis, otitis media, and multi‐allergen sensitivity. Rosmarinic acid is generally administered orally—either alone or in combination with other therapeutic agents—and is considered safe, although mild gastrointestinal side effects may occur if taken on an empty stomach. Its health‐promoting properties largely stem from its ability to neutralize free radicals and modulate inflammatory responses. Clinical investigations have emphasized its role in managing respiratory disorders exacerbated by allergic reactions and environmental pollutants (Stansbury 2014). Mechanistically, rosmarinic acid inhibits lipoxygenase and cyclooxygenase enzymes, and more recent studies reveal that it also interferes with T‐cell receptor (TCR)‐mediated signaling pathways by suppressing PLC‐γ1 and Itk activities—a mechanism similar to that of some nonsteroidal anti‐inflammatory drugs (NSAIDs) (Lee et al. 2009; Petersen and Simmonds 2003). In a phytochemical study on *Satureja hortensis* L. from the Marmara Region (Türkiye), thymol, vanillic acid, and caffeic acid were detected in ethanol extracts using TLC and LC–MS/MS methods (Akin et al. 2021). In our current analysis, caffeic acid was measured at 72.43 ng/mL in the aqueous extract of *S. macrantha* , while vanillic acid was not detected. In contrast, the methanol extract contained a notable amount of vanillic acid (4240.66 ng/mL), with caffeic acid absent. Additionally, a UHPLC‐ESI‐MS/MS‐based study on *S. boissieri* collected from Bingöl (Türkiye) reported hesperidin (5051 ± 247 ppb) and rosmarinic acid (4364 ± 214 ppb) as major constituents, attributing the plant's bioactivity to its high phenolic content (Aras et al. 2018). These findings are consistent with our results, where rosmarinic acid levels reached 50,687.02 ng/mL in the aqueous extract of *S. macrantha* , accompanied by hesperidin at 60,089.64 ng/mL. In the methanol extract, rosmarinic acid was even more concentrated (130,792.51 ng/mL), followed by hesperidin (25,749.17 ng/mL). Taken together, these findings highlight the remarkable phenolic richness of *S. macrantha* , particularly in rosmarinic acid and hesperidin, supporting its strong antioxidant and anti‐inflammatory potential.

### 5.2 Total Phenolic, Flavonoid, and Tannin Content Quantification

Phenolic compounds are abundantly found in plants and have attracted growing interest due to their notable antioxidant properties and potential protective effects against chronic diseases such as cancer and cardiovascular disorders. These bioactive molecules are common in various plant‐based foods and include a broad range of chemical classes such as simple phenols, phenolic acids (e.g., benzoic and cinnamic acid derivatives), flavonoids, lignans, coumarins, stilbenes, and tannins (Ćetković et al. 2007). In a comparative study on five *Satureja* species native to Iran, researchers examined essential oil composition, phenolic content, and antioxidant activity. The highest essential oil yield was observed in *S. mutica* (2.05%), while *S. sahendica* had the lowest (0.96%). Thymol dominated in *S. spicigera* and *S. macrantha* , whereas carvacrol was more abundant in the remaining species. Most species were rich in oxygenated monoterpenes, except *S. sahendica*. Although total phenolic content did not differ significantly, antioxidant capacity varied, reflecting differences in chemical composition. Cluster analysis distinguished *S. macrantha* and *S. spicigera* as separate groups, emphasizing their unique phytochemical profiles (Jafari et al. 2023). Another study assessed the antioxidant potential of acetone, ethanol, and water extracts from *S. hortensis* leaves and flowers using multiple assays, such as DPPH radical scavenging, lipid peroxidation inhibition, iron chelation, and hydrogen peroxide scavenging. Among all extracts, the water extract contained the highest levels of total phenolics and flavonoids, correlating with the strongest antioxidant activity. These results suggest that water extracts of *S. hortensis* could serve as a promising source of natural antioxidants and be considered for use in functional foods or nutraceuticals (Yesiloglu et al. 2013).

### 5.3 ABTS˙^+^ and DPPH˙ Cation Radical Scavenging Capacity

A study conducted on *Satureja hortensis* collected from Rize evaluated total phenolic content and antioxidant activity across three flowering stages: pre‐flowering, full‐flowering, and post‐flowering. Using the Folin–Ciocalteu method, total phenolic content ranged from 746 ± 4 to 1087 ± 44 μM, with the highest levels observed at full bloom. Antioxidant activity, assessed via the DPPH assay, showed SC50 values between 6.65 ± 0.38 and 16.10 ± 0.99 μM catechin equivalent, again peaking at the full‐flowering stage. The essential oil obtained during this stage also exhibited the highest antioxidant capacity, highlighting *S. hortensis* as a promising natural antioxidant source (Yaldiz and Camlica 2017). In our study, the water extract of *S. macrantha* (50 μg/mL) showed 28.76% ± 0.02% DPPH˙ inhibition, indicating the strongest antioxidant potential among the tested samples. By comparison, the essential oil of *S. hortensis* exhibited 4.35% ± 0.01% inhibition, while *S. macrantha* essential oil showed no measurable DPPH˙ scavenging activity. Both essential oils demonstrated considerably lower activity than standard antioxidants like α‐tocopherol and Trolox. Another comparative study assessed the antioxidant capacity of ethanolic extracts from *S. hortensis* and *Artemisia dracunculus* collected in Arak, Iran, using DPPH, ABTS, and FRAP assays. *S. hortensis* consistently exhibited stronger antioxidant effects, effectively scavenging radicals and reducing iron ions in a concentration‐dependent manner, supporting its potential pharmaceutical value in combating oxidative stress (Bahramikia et al. 2008). Consistent with these reports, our DPPH˙ assay results confirmed that extracts of *S. macrantha* —particularly the water extract—had higher radical scavenging activity than the essential oils tested. However, in the ABTS˙^+^ assay, *S. macrantha* water extract demonstrated a scavenging capacity of 52.11%, surpassing not only the other samples but also standard α‐tocopherol (11.16%), highlighting its significant antioxidant potential. The discrepancies between our findings and previous studies may be attributed to differences in plant parts used, extraction solvents, harvest timing, and geographical origin, all of which influence the concentration and composition of bioactive compounds.

### 5.4 α‐Glucosidase and α‐Amylase Enzyme Inhibition Determination

Type 2 diabetes is a widespread metabolic condition characterized by postprandial hyperglycemia (PPHG). One therapeutic approach to managing PPHG is the use of α‐amylase and α‐glucosidase inhibitors, which slow down carbohydrate digestion and delay glucose absorption, thus reducing post‐meal blood glucose spikes (Proença et al. 2022). In this study, we assessed the antidiabetic potential of *Satureja macrantha* and *S. hortensis* through in vitro α‐glucosidase and α‐amylase inhibition assays, using their water and methanol extracts as well as essential oils. At 1000 μg/mL, the water extract of *S. macrantha* demonstrated the strongest α‐glucosidase inhibition (49.36%), followed by *S. hortensis* essential oil (41.33%) and *S. macrantha* methanol extract (39.43%). Notably, all of these samples outperformed acarbose, the standard reference drug, which showed 34.98% inhibition. In contrast, none of the tested samples exhibited α‐amylase inhibitory activity. Supporting these findings, *S. hispidula* , an Algerian endemic species, was previously shown to possess strong α‐glucosidase inhibitory effects. Its aqueous extract exhibited the highest activity (IC₅₀ = 23.52 ± 6.33 μg/mL), surpassing even acarbose (IC₅₀ = 405.77 ± 34.83 μg/mL). While the ethanolic extract of *S. hispidula* showed moderate α‐glucosidase activity, it was more effective in inhibiting α‐amylase (30.34% ± 4.58%) than the aqueous extract (Haouat et al. 2022). In our case, *S. macrantha* water and methanol extracts exhibited promising α‐glucosidase inhibition, but *S. hortensis* essential oil also showed significant activity (41.33% ± 4.87%), even though the other extracts from this species were inactive. Another study on *S. barceloi* from Tunisia evaluated α‐amylase inhibition in different plant parts. The aqueous leaf extract showed the highest activity (305.61 mg EA/g), followed by stems and flowers. Notably, the ethyl acetate root extract was also effective, while the hexane extract of leaves showed minimal inhibition (Raadani et al. 2024). In contrast, no α‐amylase inhibition was observed in any of the extracts or essential oils tested in our study. These inter‐study differences may stem from variations in plant species, extraction solvents, plant parts used, or environmental and geographic factors influencing chemical composition. Overall, our findings suggest that *S. macrantha* water extract and *S. hortensis* essential oil possess notable α‐glucosidase inhibitory potential, and may be valuable candidates for further antidiabetic investigations.

### 5.5 Acetylcholinesterase (AChE) and Butyrylcholinesterase (BChE) Inhibition Assays

AD is a progressive neurodegenerative condition marked by central cholinergic system impairment. The only approved symptomatic treatments are cholinesterase (ChE) inhibitors, which help increase acetylcholine (ACh) levels in the brain. While acetylcholinesterase (AChE) is the primary enzyme responsible for ACh breakdown in healthy individuals, butyrylcholinesterase (BuChE) activity rises in AD patients, making both enzymes important therapeutic targets. Recent studies emphasize the significance of BuChE in AD progression, supporting the development of dual inhibitors, such as rivastigmine, to improve treatment outcomes (Greig et al. 2001). In our study, the anti‐Alzheimer potential of *Satureja macrantha* and *S. hortensis* was investigated via AChE and BuChE inhibition assays using their water and methanol extracts as well as essential oils. At a concentration of 1000 μg/mL, the essential oil and water extract of *S. hortensis* and the methanol extract and essential oil of *S. macrantha* each showed over 10% BuChE inhibition. In comparison, the standard drug donepezil exhibited 100% inhibition under the same conditions. In the AChE inhibition assay, tested at 100 μg/mL, the essential oil and methanol extract of *S. macrantha* and the essential oil of *S. hortensis* showed more than 10% inhibition, with the highest effect observed in *S. macrantha* essential oil (16.05%). Supporting our results, a study on *S. metastasiantha*, collected from Şemdinli (Türkiye), showed that its essential oil inhibited AChE by 30% at 200 μg/mL, while BuChE inhibition reached 30% ± 2.1%, slightly outperforming its methanol extract (Carikci et al. 2020). These findings highlight that essential oils generally exhibit stronger cholinesterase inhibition compared to polar solvent extracts, particularly in BuChE assays. Similarly, a recent investigation on *S. subspicata* from Bosnia and Herzegovina evaluated its essential oil, hot water, and methanol extracts using Ellman's method. At 1–2 mg/mL, the essential oil showed moderate AChE inhibition (72.82%–76.89%) and BuChE inhibition (27.15%–51.51%). The hot water extract had mild protective effects, while the methanol extract exhibited oxidative activity upon longer incubation (Bektasevic and Politeo 2023). In line with these studies, our findings reveal that *S. macrantha* essential oil is more effective than its extracts in AChE inhibition (16%), while *S. hortensis* essential oil demonstrates the strongest BuChE inhibitory activity (35%) among all tested samples. These results support the potential of *Satureja* species—particularly their essential oils—as natural sources of cholinesterase inhibitors with possible applications in Alzheimer's disease management.

### 5.6 Antimicrobial Activity

A study by Gomes et al. (2020) examined the antimicrobial properties and phenolic content of decoctions from *Satureja montana* and *Origanum majorana* . Both plant extracts were effective against a wide spectrum of pathogens, including gram‐positive bacteria ( *Staphylococcus aureus* , *Enterococcus faecalis* , *Streptococcus dysgalactiae* ), gram‐negative strains ( *Klebsiella pneumoniae* , *Pseudomonas aeruginosa* ), and *Candida* species. Rosmarinic acid was identified as the predominant phenolic compound, and the antimicrobial activity was attributed to disruption of microbial membrane integrity. In contrast, in our study, neither the methanol nor water extracts nor the essential oil of *S. macrantha* showed activity against the tested gram‐positive or gram‐negative bacteria. *S. hortensis* essential oil, however, demonstrated activity against *S. aureus* and *E. faecalis* , though it was ineffective against *S. dysgalactiae* , *K. pneumoniae* , and *P. aeruginosa* . The water extract of *S. hortensis* did not show any antimicrobial effect. Another study on *S. bachtiarica*, collected from Shahrekord, Iran, revealed that its ethanol extract exhibited strong inhibitory effects against *E. coli* and *S. aureus* . The aqueous extract, however, was less effective. MIC and MBC values confirmed the superior efficacy of the ethanol extract, especially against *S. aureus* (Sureshjani et al. 2013). In our findings, *S. hortensis* essential oil formed a 10 mm inhibition zone against *S. aureus* and *E. coli* , while *S. macrantha* essential oil showed an 8 mm effect against *S. aureus* . Skocibušić et al. (2006) evaluated *S. subspicata* essential oil for antimicrobial activity and reported significant effects against 13 bacterial and 9 fungal strains, with MIC values ranging from 0.09 to 25.00 μL/mL. In our study, only bacterial strains were tested. *P. aeruginosa* remained unaffected by any extract. However, *S. hortensis* essential oil was effective against *E. coli* and *Acinetobacter baumannii* . Among gram‐positive bacteria, the strongest response was observed against *Bacillus cereus* , where *S. hortensis* essential oil produced a 10 mm inhibition zone. *S. macrantha* essential oil and water extract showed 7 and 6 mm zones, respectively. Additionally, *E. faecium* responded only to *S. hortensis* essential oil (8 mm inhibition). Cavar et al. (2008) assessed the antimicrobial activity of *S. montana* and *S. subspicata* essential oils from Bosnia and Herzegovina and found that all tested strains, including *S. aureus* , *S. epidermidis* , *E. coli* , *P. aeruginosa* , and *B. subtilis* , were sensitive. In our study, *S. macrantha* essential oil exhibited activity only against *S. aureus* , while *S. hortensis* essential oil showed inhibitory effects on both *S. aureus* and *E. coli* . These results suggest that while *Satureja* species show variable antimicrobial efficacy depending on the species and plant part used, the essential oil of *S. hortensis* consistently displayed broader activity in our study compared to *S. macrantha* .

## 6 Conclusion

This comprehensive comparative study highlights the distinct phytochemical profiles and biological properties of *Satureja macrantha* and *S. hortensis* , emphasizing their species‐specific therapeutic potentials. The water extract of *S. macrantha* exhibited superior antioxidant activity, which strongly correlated with its significantly higher levels of rosmarinic acid, hesperidin, and total phenolic, flavonoid, and tannin contents. These findings support the consideration of *S. macrantha* as a promising candidate for the development of antioxidant and anti‐inflammatory formulations. Conversely, *S. hortensis* essential oil demonstrated marked antimicrobial activity, particularly against clinically relevant strains such as *S. aureus* and *E. coli* , as well as notable inhibition of butyrylcholinesterase, suggesting its dual potential as an antimicrobial and neuroprotective agent. The divergence in essential oil composition between the two species, especially in key compounds such as γ‐terpinene, thymol, and p‐cymene, further reinforces the importance of chemotypic diversity in determining bioactivity. Although both species showed meaningful α‐glucosidase inhibition—surpassing the standard drug acarbose in some cases—the lack of α‐amylase inhibition underscores the need for further exploration of their antidiabetic efficacy and mechanism of action. Genotoxicity assessments affirmed the safety of aqueous and methanolic extracts at tested concentrations, though essential oils exhibited cytotoxic effects at higher doses, highlighting the importance of dosage optimization in future applications. Altogether, these findings underscore the critical influence of species selection, extraction solvent, and phytochemical composition in modulating biological activity. *S. macrantha* emerges as a potent antioxidant resource, while *S. hortensis* essential oil holds promise as a natural antimicrobial and cholinesterase‐inhibiting agent. Further in vivo studies, bioavailability assessments, and formulation trials are warranted to translate these in vitro findings into practical therapeutic or nutraceutical applications.

## Author Contributions

**Pardis Elmdoustazar:** conceptualization (equal), data curation (equal), formal analysis (equal), investigation (equal), methodology (equal), writing – original draft (equal). **Bilge Aydın:** conceptualization (equal), data curation (equal), formal analysis (equal), investigation (equal), methodology (equal), visualization (equal). **Mehmet Önal:** conceptualization (equal), data curation (equal), investigation (equal), resources (equal). **Hafize Yuca:** conceptualization (equal), data curation (equal), formal analysis (equal), investigation (equal), software (equal). **Mehmet Karadayı:** conceptualization (equal), data curation (equal), formal analysis (equal), investigation (equal). **Yusuf Gülşahin:** conceptualization (equal), data curation (equal), formal analysis (equal), investigation (equal), software (equal). **Betül Demirci:** conceptualization (equal), data curation (equal), formal analysis (equal), investigation (equal), software (equal). **Songül Karakaya:** conceptualization (equal), data curation (equal), formal analysis (equal), investigation (equal), methodology (equal), supervision (equal), writing – original draft (equal), writing – review and editing (equal). **Zühal Güvenalp:** conceptualization (equal), data curation (equal), investigation (equal), resources (equal), supervision (equal), writing – original draft (equal).

## Disclosure

The authors have nothing to report.

## Ethics Statement

The authors have nothing to report.

## Conflicts of Interest

The authors declare no conflicts of interest.

## Data Availability

The data that support the findings of this study are available on request from the corresponding author.
